# A prospective randomized study of different height of operation table for tracheal intubation with videolaryngoscopy in ramped position

**DOI:** 10.1186/s12871-022-01929-6

**Published:** 2022-12-07

**Authors:** Dongho Kang, Hong-Beom Bae, Yun Ha Choi, Joon-suk Bom, Joungmin Kim

**Affiliations:** 1 Department of Anesthesiology and Pain Medicine, Chonnam National University Hwasun Hospital, Hwasun, Chonnam, Korea; 2 Department of Anesthesiology and Pain Medicine, Chonnam National University Medical School, 160, Baekseo-ro, Dong-gu, Gwangju, 61469 Korea; 3 Department of Anesthesiology and Pain Medicine, Chonnam National University Hospital, Kwangju, Korea; 4 Department of Nursing, Honam University, Gwangju, Korea

**Keywords:** Airway management, Anesthesia, Intubation, Laryngoscopy, Patient position

## Abstract

**Background:**

Previous studies have reported that the ramped position provides a better laryngoscopic view, reduces tracheal intubation time, and increases the success rate of endotracheal intubation. However, the patient’s head height changes while in the ramped position, which in turn changes the relative positions of the patient and intubator. Thus, making these changes may affect the efficiency of tracheal intubation; however, few studies have addressed this problem. This study analyzed intubation time and conditions during tracheal intubation using videolaryngoscope in the ramped position.

**Methods:**

This prospective study included 144 patients who were scheduled to receive general anesthesia for surgeries involving orotracheal intubation. The participants were randomly allocated to either the nipple or umbilical group according to the table height. Mask ventilation was assessed using the Warters grading scale. Tracheal intubation was performed using a McGrath MAC laryngoscope. The total intubation time, laryngoscopy time, tube insertion time, and difficulty of intubation (IDS score) were measured.

**Results:**

The umbilical group had a significantly shorter laryngoscopy time (10 ± 3 vs. 16 ± 4 s), tube insertion time (18 ± 4 vs. 24 ± 6 s), and total intubation time (28 ± 5 vs. 40 ± 7 s) compared to the nipple group. No significant difference in the difficulty of mask ventilation was observed between the two groups. The IDS score was higher in the nipple than umbilical group.

**Conclusion:**

The lower (umbilical) table level reduced the intubation time and difficulty of videolaryngoscopy compared to the higher (nipple) table level.

**Trial registration:**

This study was registered at KCT0005987, 11/03/2021, Retrospectively registered.

## Background

Many studies have been conducted to determine the optimal patient position during tracheal intubation[1–6]. The sniffing position is traditionally better for direct laryngoscopy because it aligns the oropharyngeal-larynx axis of the patient. However, the head-elevated (ramped) or 25° backup positions provides favorable conditions for direct laryngoscopy of obese patients [3]. In addition, if the intubator’s line of sight is aligned on the airway axis using the ramped or 25° back-up position, the conditions are better for direct laryngoscopy and endotracheal intubation in all patients[5, 6].

The role of videolaryngoscopy during difficult airway intubation is emphasized in several guidelines[7, 8]. Furthermore, previous studies have shown that video-assisted laryngoscopy improves the laryngeal view, reduces the rate of esophageal intubation, and is associated with a higher likelihood of first-attempt intubation compared to direct laryngoscopy in the general population [9, 10]. These results suggest that videolaryngoscopy is an excellent alternative to direct laryngoscopy in patients for whom difficult intubation is expected, as well as most other patients.

The ramped position, a uniquely designed pillow is placed under the upper body and head so that the patient’s external auditory meatus is level with the sternal notch[3, 11]. Usually, when the upper body is raised by 20–25 degrees, the two points are aligned horizontally[11]. The ramped position improves the laryngeal view of direct laryngoscopy in obese patients[3], but the ramped position provides a better laryngoscopic view, reduces tracheal intubation time, and increases the success rate of endotracheal intubation in general patients[1, 2, 5, 6]. This change in position results in a closer distance between the operator and patient’s head unless the operating table height is adjusted. The high position of the patient’s head and close proximity of the operator thereto allows the glottic opening to easily and comfortably enter the operator’s field of view, which facilitates tracheal intubation during direct laryngoscopy [4–6]. Also, it has been reported that the ramped position increases the success rate of tracheal intubation and provides a better laryngeal view in patients who are expected to have difficulty in tracheal intubation[12].

It has been reported that a high table height is useful for endotracheal intubation with conventional direct laryngoscopy in the sniffing position[4]. However, there was no difference in intubation time according to table height during direct laryngoscopy intubation in ramp position[12]. Because the method of securing the glottic view is different between the direct laryngoscopy and the videolaryngoscopy, we hypothesized that ramped position changed the relation of intubator and patient head position so the intubation time using videolaryngoscope would be different depending on the table height. Thus, in this study, we investigated the effect of table height on intubation time during endotracheal intubation using a videolaryngoscope in the ramped position.

## Materials and methods

### Study design

This prospective randomized study was approved by the Institutional Review Board of Chonnam National University Hospital (CNUH-2021-052) and registered at the Clinical Research Information Service of the Korea National Institute of Health, Republic of Korea (KCT0005987). The study was conducted between March 2021 and February 2022. Written informed consent following the principles of Declaration of Helsinki was obtained from all participants before enrollment.

### Patient selection and enrolment

Patients aged 20–80 years with an American Society of Anesthesiologists classification I-III, and who were scheduled to receive general anesthesia with orotracheal intubation, were eligible for the study. Patients with a body mass index ≥ 30 kg/m^2^, or the need for awake or nasotracheal intubation, as well as those with an oropharyngeal abnormality, cervical instability, with dental problem or height less than 150 cm were excluded. The preoperative airway evaluation was conducted on the day before surgery and included measurement of the neck circumstance, sternomental distance, thyromental distance, inter-incisor distance, and Mallampati class.

### Group allocation and data collection

The patients were divided into two groups based on the table height (umbilical or nipple level). The group allocation was based on a computer-generated list of random numbers. The operating table height for the umbilical group was adjusted so that the patient’s forehead was located at the anesthesiologist’s umbilical level. The operating table height for the nipple group was adjusted so that the patient’s forehead was located at the anesthesiologist’s nipple level.

Upon arrival in the operating room, the patient is placed in a ramped position and provided with basic monitoring. (electrocardiogram, pulse oximetry, and non-invasive blood pressure). After monitoring, the assignment group was informed to the endotracheal intubator and the bed height was subsequently adjusted. All patients were preoxygenated with 100% oxygen for 3 min, and anesthesia was then induced with propofol and remifentanil. After loss of consciousness, rocuronium (0.6 mg/kg) was administered and manual mask ventilation was performed to achieve a target tidal volume of 5 ml/kg. Peak inspiratory pressure and tidal volume were monitored during mask ventilation. Two minutes after administering the rocuronium, mask ventilation was assessed using the Warters grading scale (Table 1)[13]. Subsequently, tracheal intubation was performed using the McGrath MAC video laryngoscope (Medtronic, Dublin, Ireland). The internal diameter of the endotracheal tube was 8 mm in males and 7 mm in females, and the stylet was preshaped to resemble the curvature of the McGrath MAC with a #3 blade.

**Table 1:** Warters grading scale for mask ventilation

Description/Definition	Points
Oral or nasal airway	1
PIP 20–25 cmH_2_O	1
PIP 26–30 cmH_2_O	2
PIP > 30 cmH_2_O	3
Unable to generate PIP > 30 cmH_2_O	3
Two-person ventilation	2
Tidal volume 2-5 ml/kg	2
Unable to ventilate	4

The grading system is based on the ability to achieve a target volume of 5 ml/kg (ideal body weight). *PIP* Peak inspiratory pressure

The total intubation time was calculated as the sum of the laryngoscopy time and time to insert the tube. The laryngoscopy time was defined as the time between touching the blade tip to the lip and identifying the best glottic view on the videolaryngoscope monitor. The tube insertion time was defined as the time between inserting the endotracheal tube into the mouth and the passage of the tube through the glottis. Laryngoscopy time and tube insertion time were measured by another investigator using a stopwatch. Intubator informed the intubation phase to another investigator. Intubation difficulty was assessed using the Intubation difficulty score (IDS)[14]. IDS were used to describe the difficulty when performing conventional laryngoscopy, but it is also used when using videolaryngoscope[15]. In IDS, The lifting force applied during laryngoscopy was defined as follows. Score = 0 if little effort is necessary, and score = 1 if subjectively increased lifting force is necessary[14]. All mask ventilation and tracheal intubations were performed by a single anesthesiologist with more than 5 years of experience using videolaryngoscope to reduce the difference between intubators. Intubator was a male of 175 cm in height and 70 kg in weight, and had no musculoskeletal disorders including shoulder or spine disease. Also there was no limitation in shoulder range of motion and Multidimensional Task Ability Profile. Hence, blinding was not implemented for intubation and assessment.

### Sample size justification

The number of samples was calculated using G-power 3.1.9.2 as a result of a preliminary study in which 10 people were assigned to each group. Mean value of total intubation times at the umbilical and nipple levels were 30.2 and 28.3 s, with standard deviations (SDs) of 4.4 and 3.1, respectively. A minimum of 128 samples were required to achieve 80% power and a significance level of α = 0.05. We included 144 patients considering the possibility of 10% dropouts.

### Statistical analyses

The primary outcome of this study was total intubation time, and the secondary outcomes were the Warters grading scale score (difficulty of mask ventilation) and IDS score (difficulty of tracheal intubation). The Kolmogorov-Smirnov test was performed to examine the normality of the data. Student’s *t*-test was used to compare normally distributed continuous variables, and the Mann–Whitney *U* test was used to compare non-normally distributed continuous and ordinal variables. Categorical variables were compared using the chi-square test or Fisher’s exact test. All values are presented as the number of patients, mean ± SD, or median (25–75% interquartile range). A P-value < 0.05 was considered significant.

## Results

**Fig. 1:**
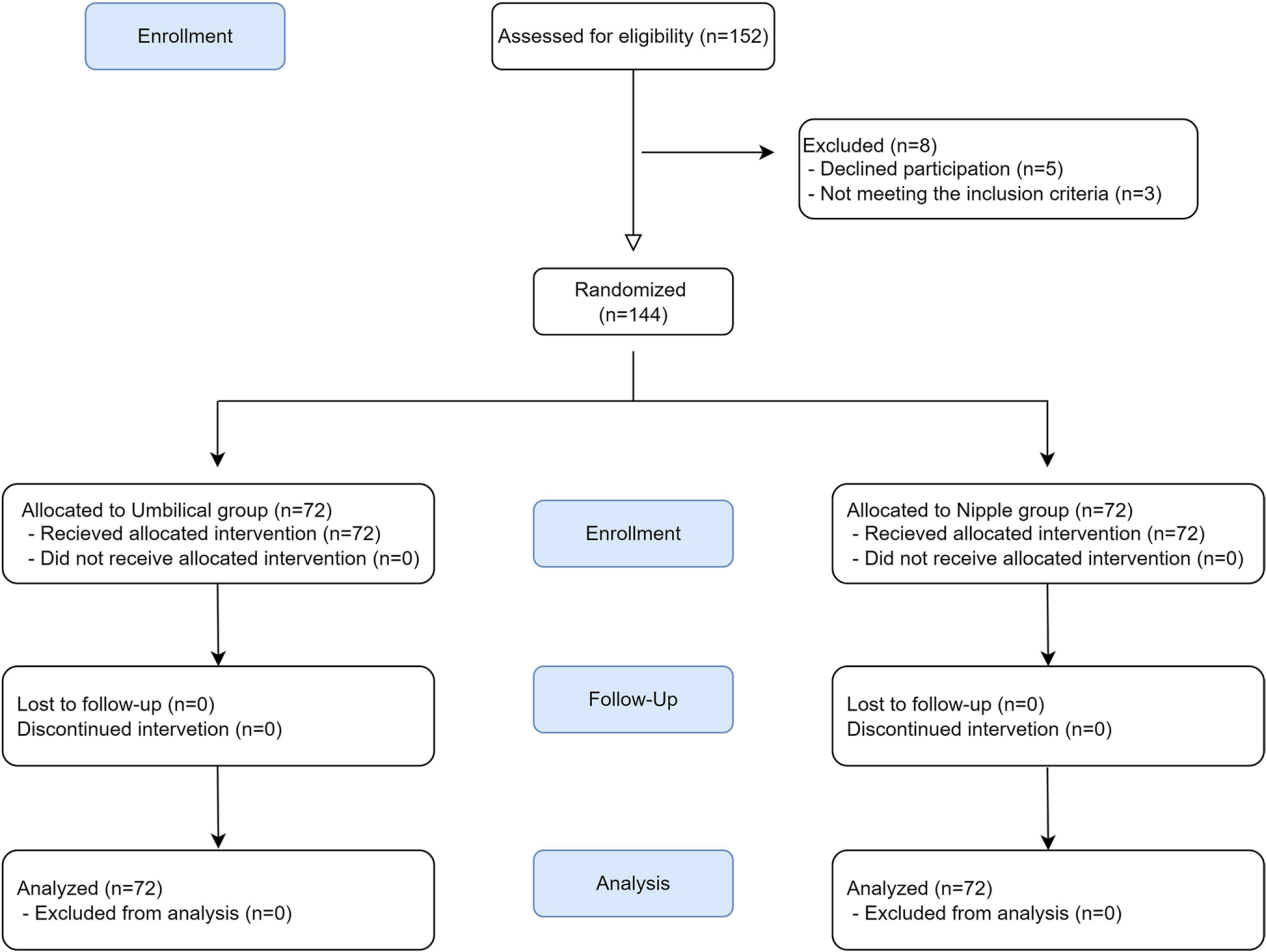
Flow diagram of the study

**Fig. 2:**
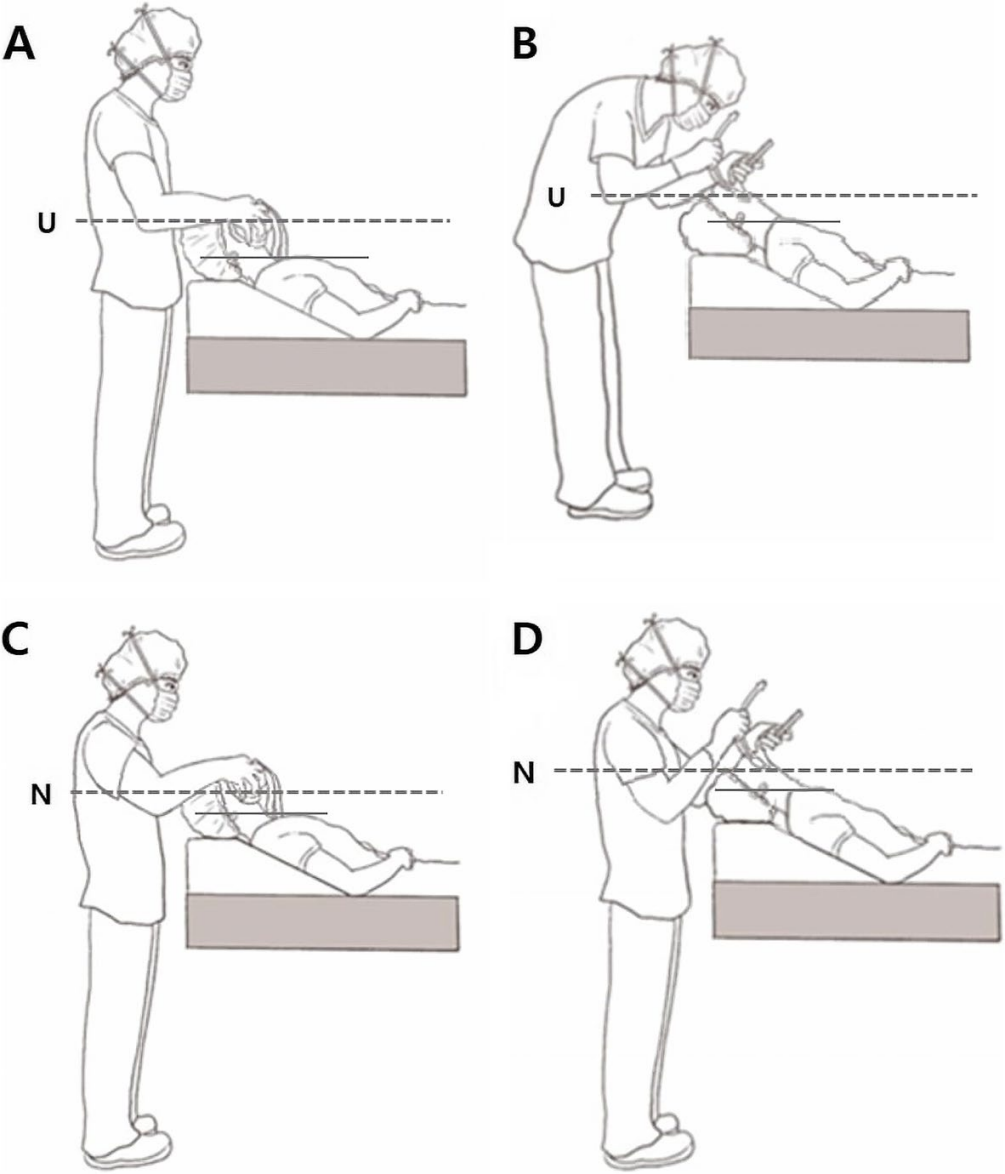
Illustration of the relation of table height and intubator. **A** Mask ventilation at umbilical table level (U). **B** Tracheal intubation at umbilical table level (U). **C** Mask ventilation at nipple table level (N). **D** Tracheal intubation at nipple table level (N)

In total, 152 patients were screened for eligibility. Eight patients were excluded for the reasons given in Fig. 1. Thus, 144 patients were included in the study. The postures during mask ventilation and endotracheal intubation for each group are shown in Fig. 2.

None of the patient characteristics or airway parameters were significantly different between the two groups, except weight and neck circumference (Table 2).

**Table 2:** Demographic data

	Nipple group (*n* = 72)	Umbilical group (*n* = 72)	*P* value
Sex (M/F)	40/32	32/40	0.182
Age (year)	52 ± 15	52 ± 14	0.891
Height (cm)	164.8 ± 7.6	162.8 ± 8.2	0.133
Weight (kg)	66.6 ± 9.3	62.7 ± 9.4	0.014
BMI (kg/m^2^)	24.5 ± 2.7	23.7 ± 3.0	0.079
ASA physical status (I/II/III)	7/49/16	10/47/15	0.740
Airway parameters			
Mallampati score (I/II/III)	62/10/0	62/9/1	0.591
Neck circumference (cm)	37.7 ± 3.1	36.2 ± 3.6	0.007
Sternomental distance (cm)	15.2 ± 3.2	15.5 ± 2.6	0.591
Thyromental distance (cm)	8.9 ± 2.7	8.3 ± 2.4	0.169
Inter-incisor distance (cm)	5.1 ± 0.8	4.9 ± 0.7	0.098

Values are presented as the number of patients or mean ± SD

The umbilical group had a significantly shorter laryngoscopy time (10 ± 3 vs. 16 ± 4 s), tube insertion time (18 ± 4 vs. 24 ± 6 s), and total intubation time (28 ± 5 vs. 40 ± 7 s) compared to the nipple group (Table 3). Neither group included cases of failed mask ventilation or intubation. A score of ≥ 4 on the Warters scale was considered to reflect difficult mask ventilation. No significant difference in the Warters scale score was observed between the two groups (Table 4).

**Table 3:** Intubation time

	Nipple group (*n* = 72)	Umbilical group (*n* = 72)	*P* value
Laryngoscopy time (s)	16 ± 4	10 ± 3	< 0.001
Tube insertion time (s)	24 ± 6	18 ± 4	< 0.001
Total intubation time (s)	40 ± 7	28 ± 5	< 0.001

Laryngoscopy time pertains to the use of a videolaryngoscope to view the glottic opening; tube insertion time refers to advancement of the endotracheal tube from the oropharynx into the trachea; total intubation time = laryngoscopy time + tube insertion time. Data are expressed as mean ± SD

**Table 4:** Difficulty of mask ventilation and tracheal intubation

	Nipple group (*n* = 72)	Umbilical group (*n* = 72)	*P* value
Difficulty of mask ventilation			
Warters scale score	1 [1–2]	1 [1–2]	0.604
No. of difficult cases (score ≥ 4)	0 (0%)	0 (0%)	NA
Difficulty of intubation			
Intubation difficulty score (IDS)	1 [0–1]	0 [0–0]	< 0.001
A. No. of attempts (n − 1)			
1	72 (100%)	72 (100%)	NA
2	0 (0%)	0 (0%)	
B. No. of operators (n − 1)			
1	72 (100%)	72 (100%)	NA
C. No. of alternative techniques (n)			
0	71 (98.6%)	71 (98.6%)	NA
1	1 (1.3%)	1 (1.3%)	
D. Cormack grade (grade - 1)			
1	66 (91.7%)	69 (95.8%)	0.457
2	5 (6.9%)	3 (4.2%)	
3	1 (1.3%)	0 (0%)	
E. Lifting force required (1)	48 (66.7%)	6 (8.3%)	< 0.001
F. External laryngeal pressure (1)	15 (20.8%)	0 (0%)	< 0.001
G. Adducted vocal cords (1)	1 (1.3%)	1 (1.3%)	NA
Ease of intubation			
Easy (IDS = 0)	21 (29.2%)	63 (87.5%)	< 0.001
Slight difficulty (0 < IDS ≤ 5)	51 (70.8%)	9 (12.5%)	
Moderate to major difficulty (IDS >5)	0 (0%)	0 (0%)	

Intubation difficulty score = sum of scores of the seven variables (A–G). An intubating stylet was used for every videolaryngoscopy, so was not considered an alternative technique. Data are expressed as number of patients (%) or median [25–75% interquartile range (IQR)].

The IDS score, which indicates the difficulty of intubation, was higher in the nipple group (1 [0–1] vs. 0 [0]), particularly for the ”lifting force required” and ”external laryngeal pressure” (Table 4). No complications related to tracheal intubation were observed in either group.

## Discussion

In this study, tracheal intubation times were compared between two table heights when using a videolaryngoscope in the ramped position. The laryngoscopy, tube insertion, and total intubation times were significantly shorter in the umbilical than nipple group. Additionally, the IDS score was lower in the umbilical than nipple group. In this study, the difference in the total intubation time of 12 s between the two groups may not be clinically significant. However, this difference is expected to be larger in obese patients taking the ramped position, as intubation conditions are often difficult.

The long laryngoscopy time of the patients in the nipple group was explained by the difficulty of insertion of the laryngoscope blade. If table height is increased, the distance between the operator and the patient’s forehead becomes shorter, so that the video laryngoscope screen and blade are not in one field of view of the intubator. The use of videolaryngoscope is associated with tooth damage[16]. Therefore, the intubator should check the monitor to determine whether the laryngoscope is in the proper position, while also taking care not to damage the lips or teeth with the laryngoscope blade. This process cannot be confirmed within one field of view of the intubator. In contrast, the intubator is relatively far from the patient’s forehead in the umbilicus group, so the screen and mouth are within the operator’s field of view; this shortens the laryngoscopy time.

The higher the table height, the more flexion and elevation of the shoulder joint occurred, which may have affected intubation time. Movements of the shoulder, elbow, and wrist joints occur during tracheal intubation. The shoulder joint mainly undergoes forward flexion but may also show elevation, rotation, or abduction. The elbow joint mainly flexes but may also supinate or pronate. When the shoulder joint flexes and elevates, movement of the abductor of the shoulder joint may be restricted due to the acromion and other surrounding structures[17]. A high table height causes the intubator’s shoulder joint to flex more than 50° and elevate. A flexed shoulder joint is more likely to cause shoulder impingement with additional shoulder joint movement[17]. In this study, limitation of joint movement during intubation using videolaryngoscope in the nipple group increased the frequency of alternative manipulation, such as “lifting force required” and “external laryngeal pressure”, which prolonged the intubation time.

Shoulder impingement causes pain and irritation in an operator with a narrow subacromial space. Repeated impingement can cause bursitis or a rotator cuff injury. Although it cannot be said that endotracheal intubation and shoulder pain are directly related, in a survey study on upper extremity pain among anesthesiologists, they complained of pain in the shoulder more frequently than in other joints (shoulder, 14%; neck, 11%; elbow, 6%; wrist and hand, 7%)[18]. Proper working posture is necessary to prevent shoulder pain in anesthesiologists. Grundgeiger et al. reported that anesthesiologists using videolaryngoscopy performed endotracheal intubation with less deflection of the joint[19]. However, a high table height during videolaryngoscopy induces shoulder impingement, making the intubator uncomfortable. In contrast, when performing tracheal intubation using a direct laryngoscope, neck, back, and knee flexion occur more frequently on a lower operating Table [4]. These flexed body positions cause discomfort to the intubator. However, there is little requirement to bend the body during tracheal intubation using a video laryngoscope because the intubation is performed at a distance from the patient, without the need to look directly into the oral cavity.

This study had several limitations. First, in this study, endotracheal intubation was performed using only one McGrath MAC type among videolaryngoscope. In previous studies, it has been reported that intubation time and conditions change depending on the type of videolaryngoscope[20]. The difference in these results is presumed to be because the shape of the blade, the angle of the camera, and the relationship between the handle and the monitor are slightly different for each product. Second, we did not measure all joint angle movements of the operator during tracheal intubation. However, previous results support that a high table position increases joints deflection during laryngoscopy. Third, only one experienced attending anesthesiologist performed the endotracheal intubations. This approach improved the reliability of the results, but it is difficult to generalize them given that there was only one experimenter. Differences in proficiency between intubators during tracheal intubation may affect outcomes, and the length of the upper limb, range of motion of the joint, and shape and length of the subacromial space differ among patients. Additional studies including more intubators with various skill levels are needed. Fourth, we did not analyze intubator comfort given that there was only one experimenter; further research on is thus necessary.

In conclusion, the present study showed that the intubation time and condition were improved when the patient’s head was placed at the umbilicus rather than the nipple level during video laryngoscopy intubation in ramped position.

## Data Availability

The datasets generated and/or analysed during the current study are not publicly available due to Hospital internal regulations but are available from the corresponding author on reasonable request.
